# Integrative taxonomy reveals new, widely distributed tardigrade species of the genus *Paramacrobiotus* (Eutardigrada: Macrobiotidae)

**DOI:** 10.1038/s41598-023-28714-w

**Published:** 2023-02-07

**Authors:** Pushpalata Kayastha, Daniel Stec, Łukasz Sługocki, Magdalena Gawlak, Monika Mioduchowska, Łukasz Kaczmarek

**Affiliations:** 1 Department of Animal Taxonomy and Ecology, Faculty of Biology, Adam Mickiewicz University in Poznań, Poznań, Uniwersytetu Poznańskiego 6, 61-614 Poznań, Poland; 2 Institute of Systematics and Evolution of Animals, Polish Academy of Sciences, Sławkowska 17, 31-016 Kraków, Poland; 3 Department of Hydrobiology, Institute of Biology, University of Szczecin, Szczecin, Poland; 4 The Institute of Plant Protection-National Research Institute, Węgorka 20, 60-318 Poznań, Poland; 5 Department of Evolutionary Genetics and Biosystematics, Faculty of Biology, University of Gdańsk, Gdańsk, Wita Stwosza 59, 80-308 Gdańsk, Poland

**Keywords:** Biodiversity, Molecular ecology

## Abstract

In a moss sample collected in Ribeiro Frio, Madeira, *Paramacrobiotus gadabouti* sp. nov. was found and described using the integrative taxonomy approach. The new species is described based on morphological and morphometric data from both phase-contrast light microscopy (PCM), as well as scanning electron microscopy (SEM). Moreover, four DNA markers, three nuclear (18S rRNA, 28S rRNA, ITS-2) and one mitochondrial (COI) markers, were used to elucidate the phylogenetic position of the new species within the family Macrobiotidae. The new species has a microplacoid that placed it within *Parmacrobiotus richtersi* group and exhibit *richtersi-*type eggs having processes terminated with cap-like structures. *Paramacrobiotus gadabouti* sp. nov. is most similar to *Pam. alekseevi*, *Pam. filipi* and *Pam. garynahi*, but differs from them mainly in details of egg morphology and morphometrics. Unlike other species from this group, which were confirmed as bisexual and showed limited distribution, *Paramacrobiotus gadabouti* sp. nov. is yet another parthenogenetic species with a wide distribution, demonstrating that at least some tardigrades confirm to the hypothesis of 'everything is everywhere'.

## Introduction

The Phylum Tardigrada currently consists of over 1400 species^1–4^ that inhabit terrestrial and aquatic (freshwater and marine) environments throughout the world^5–7^. Knowledge of terrestrial tardigrades of Madeira, Portugal is rather poor as to date, only 33 species (22 Eutardigrada and 11 Heterotardigrada taxa) have been reported from this region^8–10^.

The genus *Paramacrobiotus* Guidetti et al.^11^, currently comprises 43 formally named species^4^. It was formally erected in 2009 based on morphological and genetic analyses^11^. Morphologically two distinct species groups are present in the genus, one exhibiting a microplacoid within the pharynx, i.e. *richtersi* group and the second one without microplacoid, i.e. *areolatus* group. This phenotypic difference led Kaczmarek et al.^12^ to propose these two groups to constitute separate subgenera for which specific names were clarified by Marley et al.^13^. However, their erection was subseqently questioned independently based on two phylogenetic analyses^14,15^. Within the genus *Paramacrobiotus* bisexual and unisexual species/populations have been observed and reported in the past (e.g. in populations of *Pam. richtersi* (Murray, 1911)^16^ from Italy (bisexual and unisexual); according to modern taxonomy they probably constitute distinct species), *Pam. areolatus* (Murray, 1907)^17^ from Italy (bisexual) and Svalbard (unisexual), *Pam. tonolli* (Ramazzotti, 1956)^18^ (bisexual) from USA, *Pam. fairbanksi* Schill, Förster, Dandekar and Wolf, 2010^19^ (unisexual) from Antarctic, Italy, Poland, Spain and USA, *Pam. kenianus* Schill, Förster, Dandekar and Wolf, 2010^19^ (unisexual) from Kenya and *Pam. palaui* (unisexual) Schill, Förster, Dandekar and Wolf, 2010^19^ from Micronesia, *Pam. depressus* Guidetti, Cesari, Bertolani, Altiero and Rebecchi, 2019^14^ (bisexual) from Italy, *Pam. celsus* Guidetti, Cesari, Bertolani, Altiero and Rebecchi, 2019^14^ (bisexual) from Italy, *Pam. spatialis* Guidetti, Cesari, Bertolani, Altiero and Rebecchi, 2019^14^ (bisexual) from Italy, *Pam. arduus* Guidetti, Cesari, Bertolani, Altiero and Rebecchi, 2019^14^ (bisexual) from Italy, *Pam. experimentalis* Kaczmarek, Mioduchowska, Poprawa and Roszkowska, 2020^20^ (bisexual) from Madagascar^15,19–29.^ Importantly, Guidetti et al.^14^ also concluded that unisexual species like *Pam. fairbanksi* have a wider geographical range compared to bisexual *Paramacrobiotus* taxa. Subsequetly, Stec et al.^15^ corroborated this hypothesis additionally suggesting that the wide distribution of some taxa of the genus may be caused by human-mediated dispersion, since most of these populations were found in populated areas with trade and touristes.

In the present paper, we provide a description of a new parthenogenetic and widespread *Paramacrobiotus* species based on its population discovered in Madeira. The study was framed within an integrative taxonomy with detailed morphological and genetic analyses. We also conducted species molecular delimitation analyses based on all COI sequences of the genus *Paramacrobiotus* available in GenBank. Finally, we reconstructed the multilocus phylogeny of superclade II of the family Macrobiotidae (sensu Stec et al.^29^) to elucidate the phylogenetic position of the new species.

## Material and methods

### Sample processing

The moss sample was collected in Ribeiro Frio, Madeira (32°44′36.7"N, 16°54′28.0"W) in September 2019. The sample was packed in paper envelope, dried at a temperature of *ca.* 25 °C and delivered to the laboratory at the Faculty of Biology, Adam Mickiewicz University in Poznań, Poland. Tardigrades were extracted from the samples and studied following the protocol of Stec et al.^30^.

### Tardigrade culture

Specimens of a new species have been cultured continuously since February 2022. The cultures were maintained in plastic Petri dishes containing a mixture of ddH_2_O and “Żywiec Zdrój” spring water (3:1). To aid tardigrades locomotion, each Petri dish bottom was scratched with fine sandpaper. The culture was maintained in an environmental chamber (model POL ST1 BASIC) at 18 °C and fed once per week on rotifers (2 ml of culture of *Lecane inermis* (Bryce 1892))^31^. Once per week, the medium was exchanged using a sterile plastic pipette to avoid contamination. To establish the type of reproduction in the new species, 50 eggs were collected and incubated in a glass cube and inspected daily. Upon hatching, each juvenile was transferred to a single well of 24 well plates with scratched bottom. The isloated individuals were then observed and fed every week.

### Genotyping

Prior to DNA extraction, each tardigrade specimen was examined in vivo under PCM (400 × magnification). In order to obtain voucher specimens, genomic DNA was extracted from individual animals following a Chelex 100 resin (BioRad) extraction method^32^ with modifications according to Stec et al.^33^.

Two conservative nuclear ribosomal subunit genes were sequenced, i.e. 18S rRNA, 28S rRNA as well as nuclear ITS-2 (internal transcribed spacer 2) and mitochondrial COI (cytochrome C oxidase subunit I) barcode sequences. Fragments of the nuclear genes were amplified using the following primers: 18S_Tar_Ff1 (5ʹ-AGGCGAAACCGCGAATGGCTC-3ʹ) and 18S_Tar_Rr1 (5ʹ-GCCGCAGGCTCCACTCCTGG-3ʹ; Stec et al.^34^) for the 18S rRNA gene fragment; 28SF0001 (5ʹ-ACCCvCynAATTTAAGCATAT-3ʹ) and 28SR0990 (5ʹ-CCTTGGTCCGTGTTTCAAGAC-3ʹ; Mironov et al.^35^) for the 28S rRNA gene fragment; ITS–3 (5′- GCATCGATGAAGAACGCAGC.-3′) and ITS-4 (5′- TCCTCCGCTTATTGATATGC-3′; White et al.^36^) for the ITS-2 gene fragment. In turn, the COI molecular marker was amplified using universal primers: HCO2198 (5ʹ-TAAACTTCAGGGTGACCAAAAAATCA-3ʹ) and LCO1490 (5ʹ-GGTCAACAAATCATAAAGATATTGG–3ʹ; Folmer et al.^37^). All PCR reactions were performed in 20 μl volume containing 0.8 × JumpStart Taq ReadyMix (1 U of JumpStart Taq DNA polymerase, 4 mM Tris–HCl (pH 8.3), 20 mM KCl, 0.6 mM MgCl_2_, 0.08 mM of dNTP; Sigma-Aldrich), 0.4 μM of proper forward and reverse primers and *ca.* 1 ng of DNA. The PCR cycling profiles to amplify the 28S rRNA, ITS-2 and COI sequences were performed according to the protocols described in Kaczmarek et al.^20^. In turn, 18S rRNA sequences were amplified according to the protocol described in Stec et al.^34^. The reactions were performed in a BiometraTProfessional thermocycler. The PCR products were cleaned up by exonuclease I (20 U/μl, Thermo Scientific) and alkaline phosphatase FastAP (1 U/μl, Thermo Scientific). The Sanger sequencing method was carried out in both directions using the BigDyeTM terminator cycle sequencing and ABI Prism 3130xl genetic analyser (Life Technologies). In case ITS-2 gene fragment poor sequencing results have been obtained. Finally, this molecular marker was not applied in the analysis.

### Phylogenetic analysis and molecular species delimitation

Phylogenetic analyses were performed in order to establish phyletic position of the new species and reconstruct the relationships within Macrobiotidae clade II (sensu Stec et al.^29^). The data set was compiled from taxa/specimens for which DNA sequences of at least two (out of all four commonly used 18S rRNA, 28S rRNA, ITS-2, COI) molecular markers are available and suitable for concatenation (Table 1). The DNA sequences of *Macrobiotus rybaki* Vecchi & Stec, 2021^38^ and *Sisusbiotus spectabilis* Thulin, 1928^39^, and *Mesobiotus datanlanicus* Stec, 2019^40^ were used as the outgroup. The sequences were aligned using the AUTO method (for COI and ITS2) and the Q-INS-I method (for ribosomal markers: 18S rRNA and 28S rRNA) of MAFFT version 7^41,42^ and manually checked against non-conservative alignments in BioEdit. Then, the aligned sequences were trimmed to: 994 (18S rRNA), 811 (28S rRNA), 487 (ITS-2), 658 (COI) bp and concatenated using SequenceMatrix^43^. Before partitioning, the concatenated alignment was divided into six data blocks constituting three separate blocks of ribosomal markers and three separate blocks of three codon positions in COI data set. Using PartitionFinder^44^ under the Akaike Information Criterion (AIC), the best scheme of partitioning and substitution models were chosen for Bayesian phylogenetic analysis. Before running phylogenetic analysis, we also performed a substitution saturation test with DAMBE for two variable DNA fragments that were used in our analyses, namly COI and ITS2^45,46^. Bayesian inference (BI) marginal posterior probabilities were calculated for the concatenated (18S rRNA+28S rRNA+ITS-2+COI) data set using MrBayes v3.2^47^. Random starting trees were used and the analysis was run for ten million generations, sampling the Markov chain every 1000 generations. An average standard deviation of split frequencies of < 0.01 was used as a guide to ensure the two independent analyses had converged. The program Tracer v1.6^48^ was then used to ensure Markov chains had reached stationarity, and to determine the correct ‘burn-in’ for the analysis which was the first 10% of generations. The effective sample size (ESS) values were greater than 200 and the consensus tree was obtained after summarising the resulting topologies and discarding the ‘burn-in’. Maximum-likelihood (ML) tree was computed using RAxML v8.0.19^49^. Strength of support for internal nodes of ML construction was measured using 1,000 rapid bootstrap replicates. The consensus tree was viewed and visualised by FigTree v.1.4.3 available from http://tree.bio.ed.ac.uk/software/figtree. The best evolutionary models of sequence evolution selected for BI and ML analyses, as well as the results of saturation test are given in supplementary materials (SM.01). Networks of haplotypes of the new species were prepared using PopARTver.1.7 (http://popart.otago.ac.nz) with the implementation of Median-Joining method^50^ For this purpose, we used all COI and ITS-2 sequences od speciemens of the new species that were present in our phylogenetic analyses (N = 5 for ITS-2 and N = 8 for COI).

**Table 1:** Sequences used for phylogenetic analysis. Bold font indicates sequences obtained in this study.

Taxon	18S rRNA	28S rRNA	COI	ITS–2	Source
*Paramacrobiotus gadabouti* sp. nov. MD50.1	OP394210		OP394113		This study
*Paramacrobiotus gadabouti* sp. nov. MD50.2	OP394211	OP394209			This study
*Paramacrobiotus gadabouti* sp. nov. MD50.4	OP394212		OP394114		This study
*Macrobiotus rybaki*^37^	MW588029	MW588034	MW593931	MW588022	^37^
*Mesobiotus datanlanicus*^39^	MK584659	MK584658	MK578905	MK584657	^39^
*Minibiotus furcatus*^51^	FJ435746	FJ435760	FJ435802		^26^
*Minibiotus gumersindoi*^52^	FJ435748	FJ435761	FJ435803		^26^
*Minibiotus intermedius*^53^	ON005189	ON005195	ON005160		^54^
*Minibiotus ioculator*^33^	MT023998	MT024041	MT023412	MT024000	^33^
*Minibiotus pentannulatus* 1^55^	MT023999	MT024042	MT023413	MT024001	^33^
*Minibiotus pentannulatus* 2^55^	MT023999	MT024043	MT023414	MT024001	^33^
*Minibiotus* sp.	OK663227	OK663238		OK663216	^56^
*Paramacrobiotus* aff. *richtersi* AU	MH664932	MH664949	MH675999	MH666081	^15^
*Paramacrobiotus* aff. *richtersi* BR 1	MH664934	MH664952	MH676000	MH666082	^15^
*Paramacrobiotus* aff. *richtersi* BR 2			MH676001		^15^
*Paramacrobiotus* aff. *richtersi* BR 3			MH676002		^15^
*Paramacrobiotus* aff. *richtersi* FR 1	MH664935	MH664953	MH676003	MH666083	^15^
*Paramacrobiotus* aff. *richtersi* FR 2			MH676004		^15^
*Paramacrobiotus* aff. *richtersi* HU 1	MH664936	MH664954	MH676005	MH666084	^15^
*Paramacrobiotus* aff. *richtersi* HU 2			MH676006		^15^
*Paramacrobiotus* aff. *richtersi* MG 1	MH664938	MH664956	MH676008	MH666086	^15^
*Paramacrobiotus* aff. *richtersi* MG 2				MH666087	^15^
*Paramacrobiotus* aff. *richtersi* NO	MH664939	MH664957	MH676009	MH666088	^15^
*Paramacrobiotus* aff. *richtersi* NZ	MH664940	MH664958	MH676010	MH666089	^15^
*Paramacrobiotus* aff. *richtersi* PT 1	MH664944	MH664961	MH676014	MH666093	^15^
*Paramacrobiotus* aff. *richtersi* PT 2			MH676015		^15^
*Paramacrobiotus* aff. *richtersi* TN	MH664945	MH664962	MH676016	MH666094	^15^
*Paramacrobiotus* aff. *richtersi* TZ	MH664933	MH664951	MH676017	MH666095	^15^
*Paramacrobiotus arduus*^14^	MK041032		MK041020		^14^
*Paramacrobiotus areolatus*^17^	MH664931	MH664948	MH675998	MH666080	^15^
*Paramacrobiotus celsus*^14^	MK041031		MK041019		^14^
*Paramacrobiotus* cf. *klymenki* IT	MH664937	MH664955	MH676007	MH666085	^15^
*Paramacrobiotus* cf. *klymenki* PT	MH664943	MH664960	MH676013	MH666092	^15^
*Paramacrobiotus depressus*^14^	MK041030		MK041015		^14^
*Paramacrobiotus experimentalis*^20^	MN073468	MN073465	MN097837	MN073464	^20^
*Paramacrobiotus fairbanksi* PL^19^	MH664941	MH664950	MH676011	MH666090	^15^
*Paramacrobiotus filipi* 1^57^	MT261913	MT261904	MT260372		^57^
*Paramacrobiotus filipi* 2^57^			MT260373		^57^
*Paramacrobiotus lachowskae*^58^	MF568532	MF568533	MF568534	MF568535	^58^
*Paramacrobiotus metropolitanus*^59^	LC637243	LC649795	LC637242	LC649794	^59^
*Paramacrobiotus richtersi*^16^	MK041023		MK040994		^14^
*Paramacrobiotus richtersi* S38 1^16^	OK663224	OK663236	OK662995	OK663213	^56^
*Paramacrobiotus spatialis*^14^	MK041024		MK040996		^14^
*Paramacrobiotus spatialis* S107 1^14^	OK663225	OK663236	OK662996	OK663214	^56^
*Paramacrobiotus tonolli* US^18^	MH664946	MH664963	MH676018	MH666096	^15^
*Sisubiotus spectabilis*^38^	MN888371	MN888357	MN888322	MN888331	^37^
*Tenuibiotus* cf. *ciprianoi*	MN888376	MN888361	MN888328	MN888348	^37^
*Tenuibiotus danilovi*^60^	MN888377	MN888362	MN888329	MN888349	^37^
*Tenuibiotus tenuiformis*^60^	MN888378	MN888363	MN888330	MN888350	^37^
*Tenuibiotus voronkovi*^60^	KX810045	KX810049	KX810042	KX810046	^61^
*Tenuibiotus zandrae*^62^	MN443040	MN443035	MN444827	MN443038	^62^

Using the COI data set comprising all *Paramacrobiotus* sequences of this marker avilabe in GenBank (80 sequences), we performed two genetic species delimitation analyses. According to the recommendation by Fontaneto et al.^63^ one of them was a tree-based method, the Poisson Tree Processes (bPTP^64^), whereas the second one was a distance-based method, the Assemble Species by Automatic Partitioning (ASAP^65^). For the bPTP analysis, we computed a ML tree using RAxML v8.0.19^49^ also with prior search of the best model and partition scheme using PartionFinder2^66^ (SM.01). The calculations were conducted on the bPTP webserver (http://species.h-its.org/ptp), with 500,000 MCMC generations, thinning the set to 100, burning at 10% and performing a search for ML and Bayesian solutions. For ASAP analysis we used the COI alignment as input data. The analyses were run on the respective server (https://bioinfo.mnhn.fr/abi/public/asap/asapweb.html) with default settings. All COI sequences used for the analyses and their outputs are given within the supplementary materials (SM.02).

### Microscopy and imaging

In total 33 animals, 5 exuvium, 2 simplex and 24 eggs were mounted on microscope slides in the Hoyer’s medium, and then examined under Olympus BX41 Phase-contrast light Microscope (PCM) associated with Olympus SC50 digital camera (Olympus Corporation, Shinjuku-ku, Japan). Thirty animals and 10 eggs were prepared for Scanning Electron Microscope (SEM) observation according to the protocol in Roszkowska et al.^67^ and examined under high vacuum in Hitachi S3000N SEM (Hitachi, Japan). All figures were assembled in Corel Photo-Paint 2017. For deep structures that could not be fully focused on a single photograph, a series of 2–10 images were taken every *ca.* 0.5 μm and then manually assembled into a single deep-focus image in Corel Photo-Paint 2017.

### Morphometrics and morphological nomenclature

All measurements are given in micrometers [μm]. Structures were measured only if their orientation was suitable. Body length was measured from the anterior extremity to the end of the body, excluding the hind legs. The types of bucco-pharyngeal apparatuses and claws were classified according to Pilato and Binda^68^. All measurements and terminology of adults and eggs of *Paramacrobiotus* were prepared according to Kaczmarek and Michalczyk^69^ and Kaczmarek et al.^12^. Terminology describing the oral cavity armature (OCA) follows Michalczyk and Kaczmarek^70^. The macroplacoid length sequence was indicated according to Kaczmarek et al.^71^. Morphological states of the cuticular bars on legs follow Kiosya et al.^72^. The *pt* ratio is the ratio of the length of a given structure to the length of the buccal tube expressed as a percentage^73^. Morphometric data were handled using the “Parachela” ver. 1.8 template available from the Tardigrada Register^74^. Row morphometric data for the new species are given in Supplementry Materials (SM.03). Tardigrade taxonomy follows Bertolani et al.^75^ and Stec et al.^29^. Genus abbreviations follow Perry et al.^76^.

### Comparative material

For comparison with the new species, holotype and/or paratypes of *Pam. derkai* (Degma, Michalczyk and Kaczmarek, 2008)^77^, *Pam. experimentalis*, *Pam. fairbanksi* Schill, Förster, Dandekar and Wolf, 2010^19^, *Pam. filipi* Dudziak, Stec and Michalczyk, 2020^57^, *Pam. garynahi* (Kaczmarek, Michalczyk and Diduszko, 2005)^78^, *Pam. huziori* (Michalczyk and Kaczmarek, 2006)^79^, *Pam. intii* Kaczmarek, Cytan, Zawierucha, Diduszko and Michalczyk, 2014^71^, *Pam. lachowskae* Stec, Roszkowska, Kaczmarek and Michalczyk, 2018^58^, *Pam. magdalenae* (Michalczyk and Kaczmarek, 2006)^80^, *Pam. sklodowskae* (Michalczyk, Kaczmarek and Węglarska, 2006^81^) and *Pam. spinosus* Kaczmarek, Gawlak, Bartels, Nelson and Roszkowska, 2017^12^ were examined. Moreover, for species identification, the key in Kaczmarek et al.^12^ and original descriptions were also used (i.e.^15,78,82^).

## Results

### Phylogeny and species delimitation

**Figure 1:**
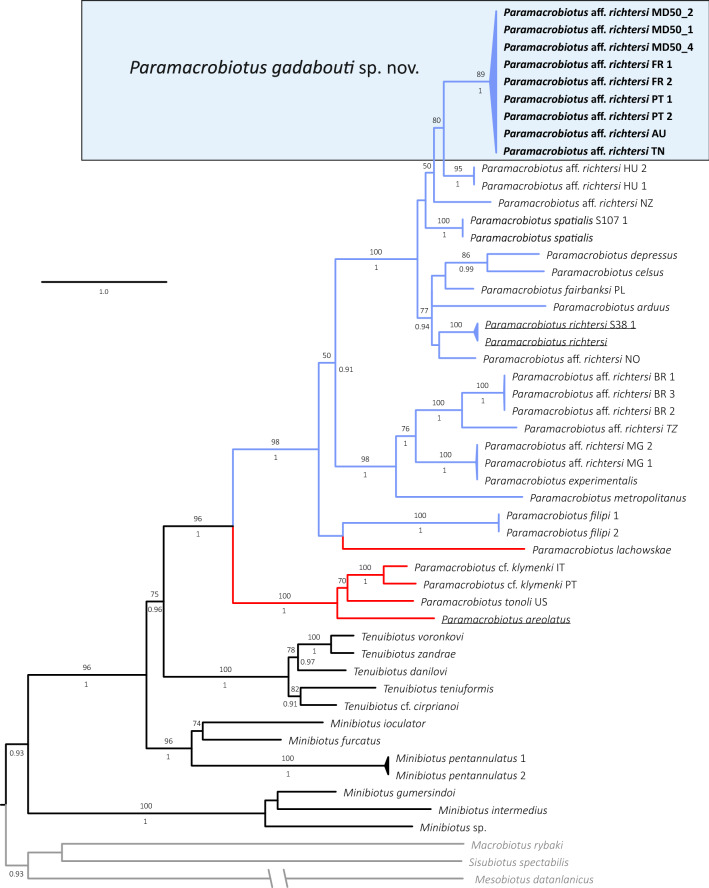
Maximum likelihood (ML) phylogeny constructed from concatenated sequences of the family Macrobiotidae (18S rRNA + 28S rRNA + ITS-2 + COI; Table 1). Numbers above branches indicate bootstrap support values, while Bayesian posterior probabilities (pp) are given below branches. Bootstrap < 50 and pp < 0.90 are not shown. Taxa of the *Pam. richtersi* and *Pam. areolatus* complex are indicated by blue and red branches, respectively. The outgroup is indicated in gray font. The scale bar represents substitutions per position.

**Figure 2:**
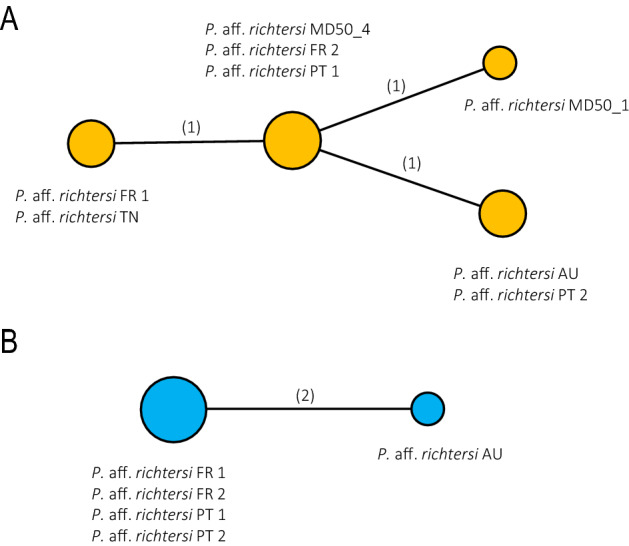
Haplotype Median Joining networks for mitochondrial (COI) and nuclear (ITS-2) markers of *P. gadabouti* sp. nov: (**A**)—COI; (**B**)—ITS-2. Haplotypes are represented by coloured circles. The size of circles is proportional to the number sequences/specimens of each particular haplotype. Sequence/specimen names correspond with names presented in phylogenetic tree in Fig. 1. Numbers in brackets indicate the numbers of mutations between the haplotypes.

Both phylogenetic analyzes resulted with trees of similar topology and mostly well-supported nodes in which *Paramacrobiotus* and *Tenuibiots* are monophyletic genera, whereas *Minibiotus* was recovered paraphyletic (Fig. 1). Monophyly was not confirmed for *Pam. richtersi* and *Pam. areolatus* morpho-groups since representatives of the latter form a paraphyletic group caused by *Pam. lachowskae* which cluster together with the former morpho-group (Fig. 1). The sequences of the new species obtained in this study clastered together with *Paramacrobiotus* aff. *richtersi* populations from France, Portugal, Australia and Tunisia previously reported by Stec et al.^15^, forming a monophyletic clade staying in sister relationship with *Paramacrobiotus* aff. *richtersi* population from Hungary. Haplotype networks showed higher haplotype diversity in case of COI than in ITS-2 marker, with same COI haplotype shered sometimes with populations from very distinct localities (Fig. 2). Molecular species delimitation analyzes recovered 22 and 29 putative species for ASAP and bPTP methods, respectively, with all valid nominal taxa delineated coherently as distinct entities (SM.02). Importantly, 9 ASAP and 12 bPTP entities were delimited from COI sequences without assignment to any nominal *Paramacrobiotus* species. Single locus delimitations confirmed the results from multilocus phylogeny, recognizing the newly studied population and *Paramacrobiotus* aff. *richtersi* populations from France, Portugal, Australia and Tunisia as a single species (Fig. 1; SM.02) which is formally described below.

### Taxonomic Account

- *Phylum*: Tardigrada (Doyère, 1840)^83^
- *Class*: Eutardigrada (Richters, 1926)^84^
- *Order*: Macrobiotoidea (Thulin, 1928)^39^
- *Family*: Macrobiotidae (Thulin, 1928)^39^
- *Genus*: *Paramacrobiotus* (Guidetti et al., 2009)^11^

**Figure 3:**
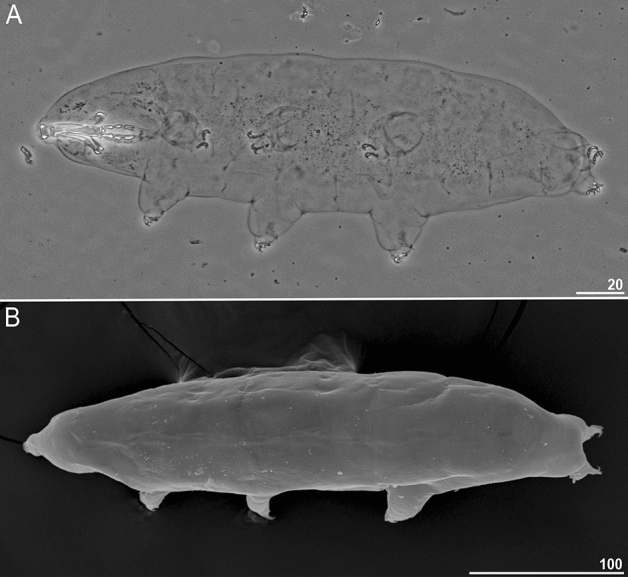
*Paramacrobiotus gadabouti* sp. nov.: (**A**)—ventral-dorsal projection (holotype, PCM); (**B**)—dorso-ventral projection (paratype, SEM). Scale bars in µm.

**Figure 4:**
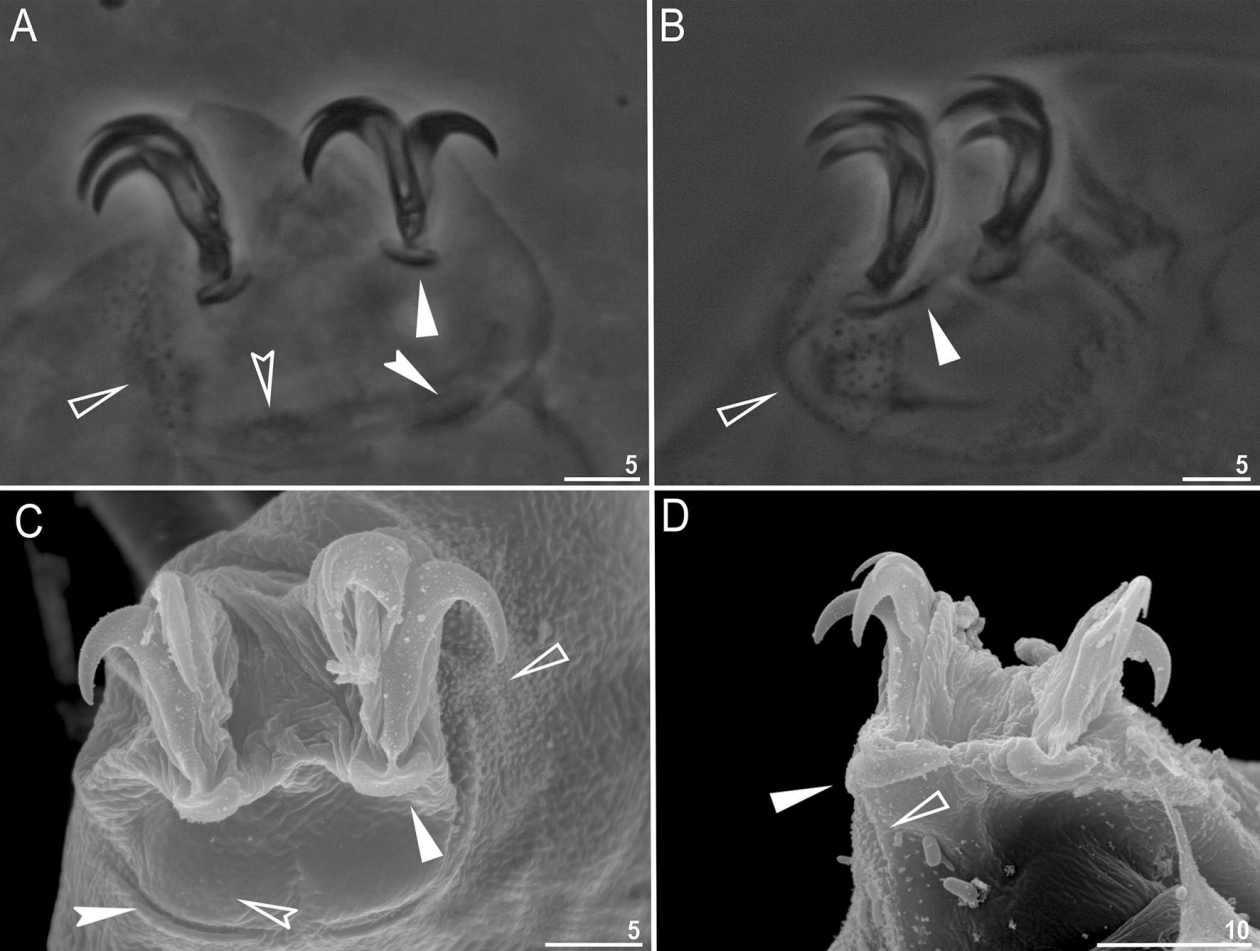
*Paramacrobiotus gadabouti* sp. nov.: (**A**)—claws II (paratype, PCM); (**B**)—claws IV (paratype, PCM); (**C**)—claws II (paratype, SEM); (**D**)—claws IV (paratype, SEM). Filled unindented arrowhead represents smooth lunulae, empty unindented arrowhead represents granulation, empty indented arrowheads represent single continuous bar and filled indented arrowheads represent doubled muscle attachments. Scale bars in µm.

**Figure 5:**
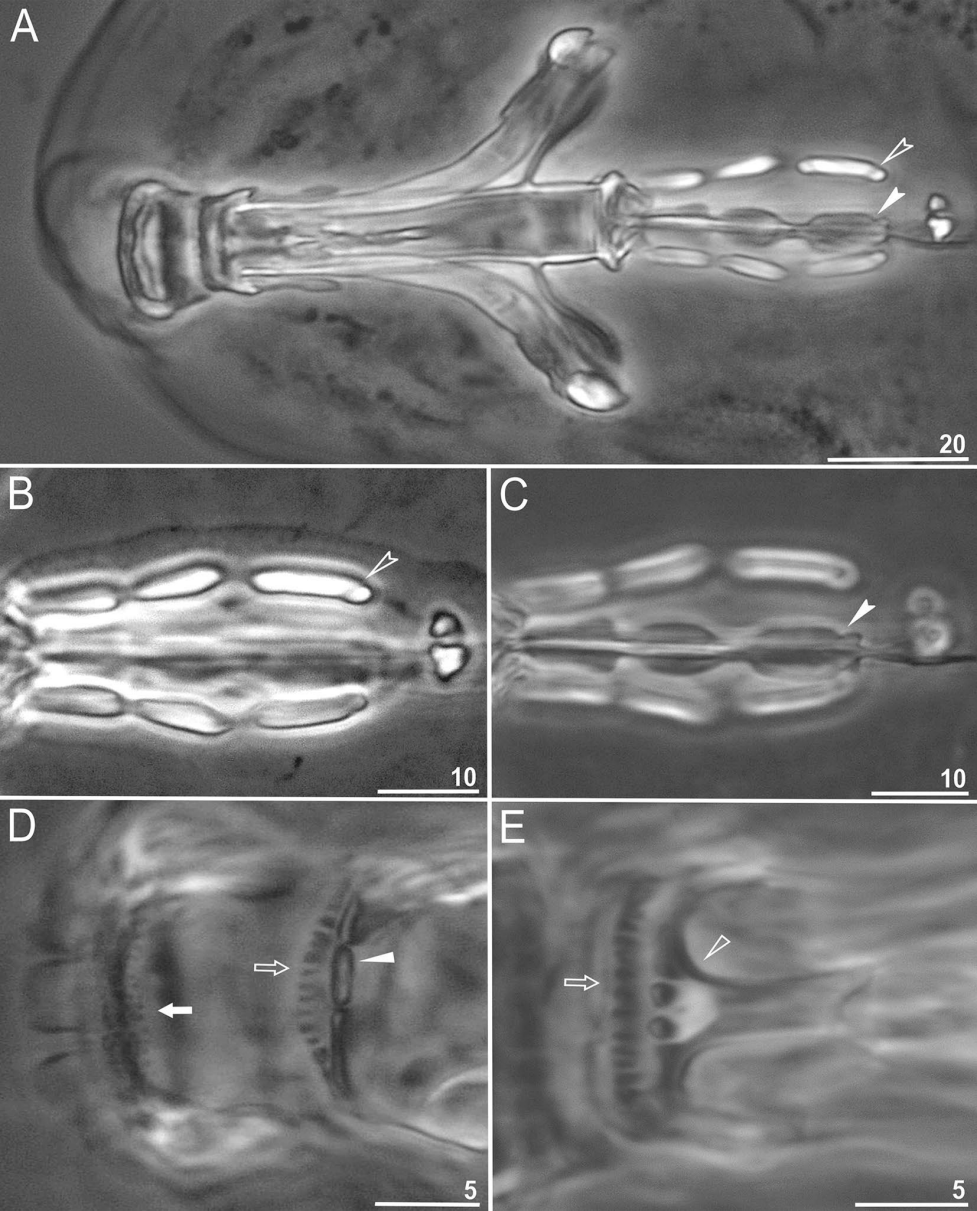
*Paramacrobiotus gadabouti* sp. nov.: (**A**)—bucco-pharyngeal apparatus (dorso–ventral projection) general view (paratype); (**B**)—placoid morphology in dorsal view (paratype); (**C**)—ventral placoids (paratype); (**D**)—oral cavity armature (paratype, PCM) seen from the dorsal side; (**E**)—oral cavity armature (paratype, PCM) seen from the ventral side. Empty indented arrowhead represents third macroplacoid with sub-terminal constriction, filled indented arrowhead represents third macroplacoid with central constriction in ventral side, filled arrow represents first band of teeth, empty arrow represents second band of teeth, filled unindented arrowhead represents third band of teeth from dorsal side and empty unindented arrowhead represents third band of teeth from ventral side. Scale bars in µm.

**Figure 6:**
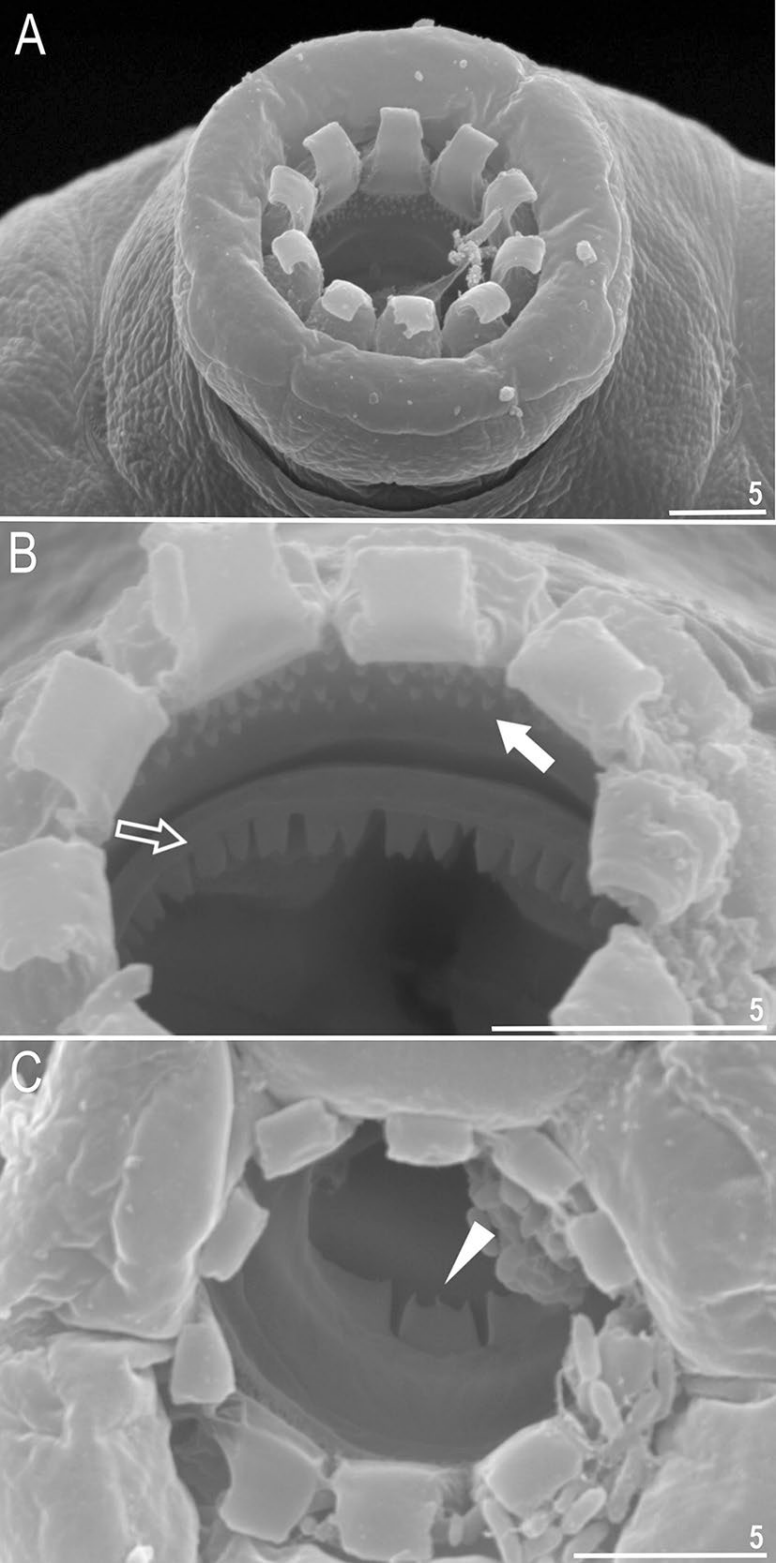
*Paramacrobiotus gadabouti* sp. nov.: (**A**)—mouth with ten peribuccal lamellae (paratype, SEM); (**B**)—oral cavity armature with first and second band of teeth (paratype, SEM); (**C**)—oral cavity armature with third band of teeth (paratype, SEM) from dorsal side. Filled arrow represents first band of teeth, empty arrow represents second band of teeth and filled unindented arrowhead represents third band of teeth. Scale bars in µm.

**Figure 7:**
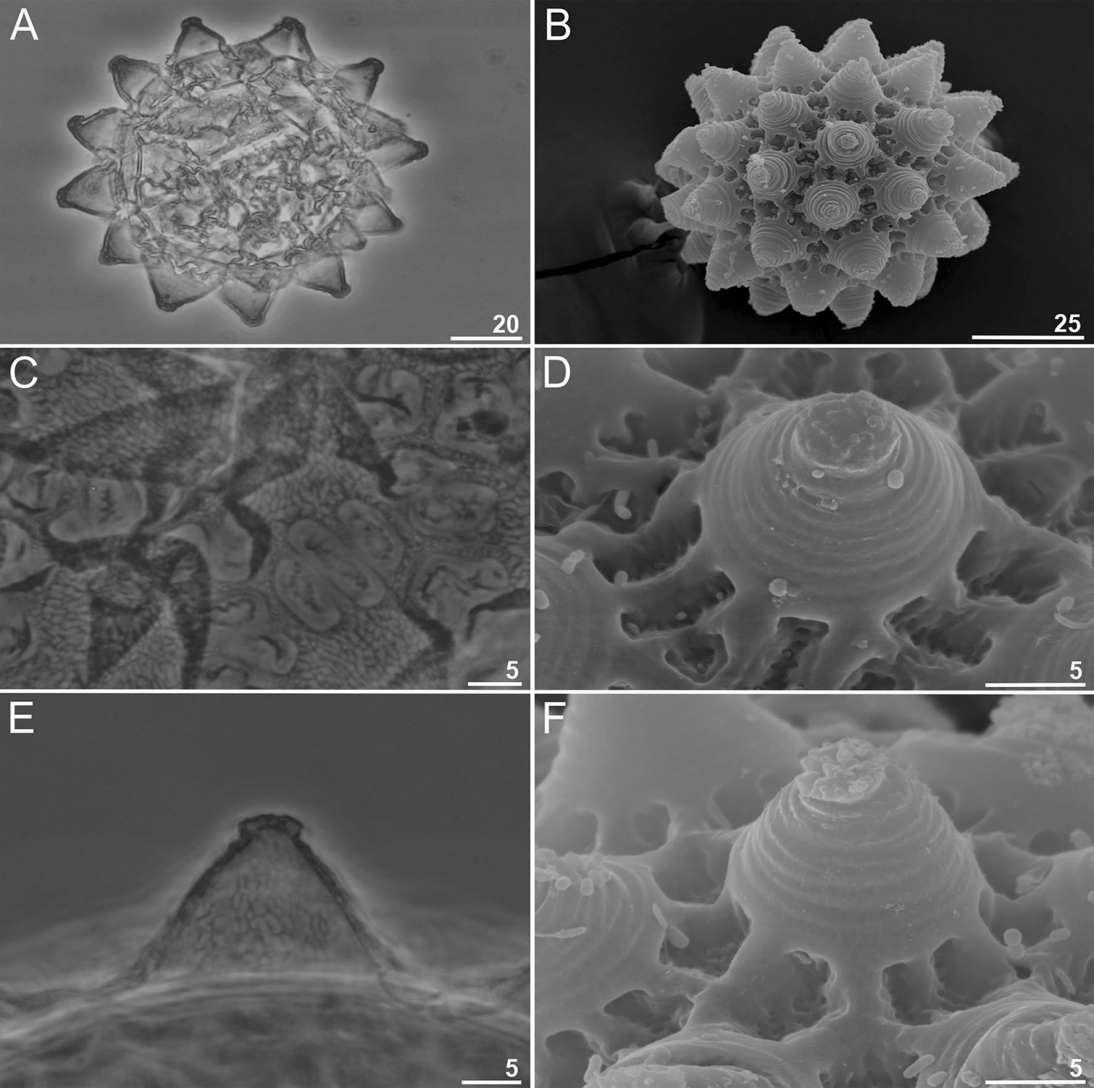
*Paramacrobiotus gadabouti* sp. nov.: eggs: (**A**, **B**)—egg chorion (paratype, PCM and SEM respectively); (**C**, **D**)—the surface between egg processes (paratype, PCM and SEM respectively); (**E**, **F**)—egg processes (paratype, PCM and SEM respectively). Scale bars in µm.

*Paramacrobiotus gadabouti* sp. nov. Kayastha, Stec, Mioduchowska and Kaczmarek.

(Figs. 2, 3, 4,5, 6 and 7; Tables 2 and 3).

**Table 2:** Measurements [in µm] and *pt* values of selected morphological structures of individuals of *Paramacrobiotus gadabouti* sp. nov. mounted in Hoyer’s medium (N—number of specimens/structures measured; RANGE refers to the smallest and the largest structure among all measured specimens; SD—standard deviation, *pt*—ratio of the length of a given structure to the length of the buccal tube expressed as a percentage).

CHARACTER	N	RANGE		MEAN		SD		Holotype	
		µm	*pt*	µm	*pt*	µm	*pt*	µm	*pt*
Body length	20	435–783		588		95		594	
Buccopharyngeal tube									
Buccal tube length	20	44.7–64.5		54.1		4.5		50.0	
Stylet support insertion point	20	36.2–49.8	*76.1–81.0*	42.4	*78.3*	3.4	*1.2*	39.7	*79.4*
Buccal tube external width	20	9.1–15.4	*20.0–27.9*	12.9	*23.7*	1.7	*1.9*	11.9	*23.7*
Buccal tube internal width	20	6.4–10.8	*14.2–19.6*	9.2	*17.0*	1.2	*1.3*	8.9	*17.7*
Ventral lamina length	17	23.9–38.6	*53.4–60.6*	30.8	*57.4*	3.6	*2.2*	28.4	*56.7*
Placoid lengths									
Macroplacoid 1	20	7.7–12.6	*17.1–21.8*	10.4	*19.1*	1.4	*1.3*	8.8	*17.6*
Macroplacoid 2	20	7.0–11.3	*15.4–19.8*	9.6	*17.7*	1.3	*1.3*	8.3	*16.5*
Macroplacoid 3	20	8.4–14.9	*18.7–26.4*	12.5	*22.9*	1.7	*1.8*	11.4	*22.8*
Microplacoid	20	3.3–5.1	*6.7–9.3*	4.2	*7.9*	0.5	*0.8*	4.7	*9.3*
Macroplacoid row	20	27.4–41.9	*60.7–69.8*	35.1	*64.7*	3.9	*2.8*	32.3	*64.6*
Placoid row	20	34.9–53.6	*77.7–87.4*	44.8	*82.6*	4.8	*3.3*	41.8	*83.5*
Claw I heights									
External primary branch	19	11.3–20.5	*24.0–31.8*	15.1	*27.6*	2.0	*1.9*	14.2	*28.4*
External secondary branch	19	9.2–17.1	*19.2–26.5*	11.8	*21.6*	1.6	*1.5*	11.6	*23.2*
Internal primary branch	19	11.7–19.1	*23.3–29.6*	14.2	*26.2*	1.7	*1.7*	13.8	*27.6*
Internal secondary branch	19	9.2–15.2	*16.9–23.6*	11.3	*20.8*	1.4	*1.6*	10.4	*20.8*
Claw II heights									
External primary branch	19	11.8–23.9	*24.5–37.1*	15.5	*28.4*	2.5	*2.8*	14.7	*29.3*
External secondary branch	19	8.1–16.2	*13.9–25.5*	11.9	*21.9*	2.1	*3.2*	11.4	*22.7*
Internal primary branch	19	11.8–20.0	*23.6–31.0*	14.5	*26.5*	1.9	*1.8*	11.8	*23.6*
Internal secondary branch	19	9.6–15.4	*19.7–24.2*	11.8	*21.7*	1.5	*1.4*	10.7	*21.4*
Claw III heights									
External primary branch	19	12.9–21.6	*26.8–33.5*	15.9	*29.3*	1.9	*1.5*	14.3	*28.6*
External secondary branch	19	9.7–15.2	*18.5–27.2*	12.3	*22.6*	1.5	*2.0*	11.1	*22.2*
Internal primary branch	19	12.1–20.3	*24.0–31.5*	14.7	*27.1*	1.8	*1.8*	14.0	*27.9*
Internal secondary branch	19	10.1–17.1	*18.6–26.5*	11.8	*21.6*	1.6	*2.0*	11.4	*22.7*
Claw IV heights									
Anterior primary branch	19	11.7–22.8	*26.2–35.4*	16.7	*30.7*	2.1	*2.2*	15.7	*31.4*
Anterior secondary branch	19	8.7–19.2	*17.7–29.8*	13.0	*23.8*	2.3	*2.9*	11.4	*22.7*
Posterior primary branch	19	13.4–23.1	*27.3–35.8*	16.3	*30.0*	2.2	*2.2*	14.3	*28.6*
Posterior secondary branch	19	10.7–17.0	*21.6–29.0*	12.9	*23.9*	1.4	*2.0*	12.6	*25.1*

*pt* values are in italic.

**Table 3:** Measurements [in µm] of selected morphological structures of eggs of *Paramacrobiotus gadabouti* sp. nov. mounted in Hoyer’s medium (N—number of specimens/structures measured; RANGE refers to the smallest and the largest structure among all measured eggs; SD—standard deviation).

CHARACTER	N	RANGE	MEAN	SD
Egg bare diameter	17	64.3–91.7	78.2	6.8
Egg full diameter	17	104.8–125.3	112.7	7.2
Process height	50	12.1–23.7	17.5	2.2
Process base width	50	15.0–25.5	19.2	2.3
Process base/height ratio	50	91%–135%	110%	12%
Inter-process distance	50	2.5–6.1	3.8	0.8
Number of processes on the egg circumference	17	11–13	12.1	0.9

*Type Material.* Holotype (slide M50/4 (+ 6 paratypes (3 animals + 2 exuvium + 1 simplex) on the same slide)) and 101 paratypes (29 animals + 3 exuvium + 1 simplex + 24 eggs; slides: M50/*, where the asterisk can be substituted by any of the following numbers: 1–3, 5–20), 4 exoskeleton after DNA extraction (M50.1/S, M50.2/S, M50.3/S and 50.4/S) and 30 animals + 10 eggs on one SEM stub.

*Description (measurements and statistics in *Table 2).* Animals:* Body colour transparent/white, eyes absent in living specimens (Fig. 3A–B). Except for granulation on legs I–IV (Fig. 4A–D), cuticle is smooth, i.e. without gibbosities, papillae, pores, spines or sculpturing. The leg granulation is present on the external surface of legs I–III and on lateral and dorsal surfaces of the hind legs (Fig. 4A–D). Claws of the *hufelandi* type, stout (Fig. 4A–D). Primary branches with distinct accessory points. Smooth lunulae present under all claws (Fig. 4A–D, filled unindented arrowhead). Single continuous cuticular bar constricted in the middle and paired muscle attachments below claws I–III present (Fig. 4A–D, empty indented arrowhead and filled indented arrowhead).

Bucco-pharyngeal apparatus of the *Macrobiotus* type (Fig. 5A–C), with ventral lamina and ten peribuccal lamellae (Fig. 6A). Mouth antero-ventral. The OCA is composed of three bands of teeth (similar on dorsal and ventral sides) (Figs. 5D–E and 6A–C). The first band of teeth consists of small cones (granules in PCM) situated at the anterior portion of the oral cavity, and just behind the base of the peribuccal lamellae (4–5 rows) (Figs. 5D, 6B, filled arrow). The second band of teeth positioned in the rear of the oral cavity between the ring fold and the third band of teeth (Figs. 5D, 6B, empty arrow) and composed of larger cones (small ridges parallel to the main axis of the buccal tube in PCM), arranged in one row that runs around the oral cavity wall (Figs. 5D–E and 6B, filled unindented arrowhead). The third band of teeth positioned just before the buccal tube opening and composed of dorsal and ventral portion (Figs. 5D–E and 6B–C). The dorsal portion of the third band comprises three, distinctly separated, long and thin ridges (Fig. 5D and 6C). Similarlty, the ventral portion is composed of three distinct teeth with two ventro-lateral onces in shape of ridges and one medio-ventral tooth being often divided into 2–3 smaller granular teeth (Fig. 5E). Additional teeth absent (Figs. 5D–E and 6A–C). Pharyngeal bulb spherical, with triangular apophyses and three rod-shaped macroplacoids. Macroplacoid length sequence 2 < 1 < 3 (Fig. 5A–C). The first macroplacoid without constrictions, but distinctly narrower anteriorly. The second macroplacoid of uniform width and without constrictions. The third macroplacoid with a sub-terminal constriction (Fig. 5A–B; empty unindented arrowhead). Microplacoid present, triangular in shape (Fig. 5A–B).

*Eggs:* Laid freely, white, spherical exhibiting ornamentations of the *richtersi* type (Fig. 7A–B). Processes in the shape of rounded or truncated cones (Fig. 7A–F). Top endings of the processes with cap like structures (well visible in PCM in the process midsection and always well visible in SEM) (Fig. 7D–F). The surface of cap-like structrues is mostly rough with small granules and wrinkles that can be visible on its surface but only in SEM (Fig. 7D, F). Labyrinthine layer between process walls visible under PCM as a clear reticular pattern (Fig. 7C). Reticular pattern composed of regular and elongated mesh with straight or slightly sinuous margins. Egg shells areolated with a single ring of 10–12 areolae around each process (Fig. 7C–D). Internal surface of areolae clearly sculptured in PCM and pores that are visible only in SEM (Fig. 7C–D).

*Reproduction:* In the experimental setting with isolated individuals of the new species, eggs laying was observed in all matured animals. These eggs hatched into juveniles. Thus, we conclude the reproduction in *Pam gadabouti* sp. nov. to be parthenogenetic.

*Type Locality*: Portugal, Madeira Island, 32°44′36.7"N, 16°54′28.0"W, 647 m asl, Ribeiro Frio, moss from rock and rock wall, 23 Sptember 2019, coll. Łukasz Sługocki, Ricardo Araújo and J. J. Gonçalves Silva.

*Additional Localities*: (1) Portugal, Madeira Island, 32°49′06″N, 16°59′19″W, 299 m asl, Ponta Delgada, moss from rock, 21 February 2018, coll. Łukasz Michalczyk; (2) Australia, Western Australia State, 31°57′16″S, 115°50′40″E, 56 m asl, Perth, Kings Park, moss from tree, 22 March 2015, coll. Łukasz Michalczyk; (3) France, Île-de-France Region, 48°51′35.5″N, 2°23′40″E, 80 m asl, Paris, Père Lachaise Cemetery, moss from grave, 23 May 2016, coll. Witold Morek; (4) Tunisia, Beni M'tir, Jendouba Governorate, 36°73′92″N, 8°72′99″E, 516 m asl, moss from soil in a forest, 12 June 2015, coll. Jamila Ben Marnissi. All these additional localities have been previously reported in Stec et al.^15^.

*Etymology*: The name *‘gadabouti’* refers to the new species ubiquity; from Eng. ‘gadabout’: someone who restlessly moves from place to place seeking amusement or the companionship of others.

*Type depositories*: Holotype (M50/4 (+ 6 paratypes (3 animals + 2 exuvium + 1 simplex) on the same slide)) and 97 paratypes (slides: M50/*, where the asterisk can be substituted by any of the following numbers: 1–3, 5–20, 1/S, 2/S, 3/S and 4/S) were deposited at the Department of Animal Taxonomy and Ecology, Institute of Environmental Biology, Adam Mickiewicz University in Poznań, Uniwersytetu Poznańskiego 6, 61–614 Poznań, Poland and four paratypes (slides: M50/7 and M50/13 (3 animals and 1 egg)) were deposited at Institute of Systematics and Evolution of Animals, Polish Academy of Sciences, Sławkowska 17, 31–016, Kraków, Poland.

## Discussion

### Differential diagnosis of the new species

*Paramacrobiotus gadabouti* sp. nov. by having a microplacoid in the pharynx and eggs ornamentation of the *richtersi* type with egg processes ended with cap-like structures is similar to *Pam. alekseevi*^82^ (Tumanov 2005), *Pam. filipi*^57^ Dudziak, Stec and Michalczyk 2020 and *Pam. garynahi*^78^ (Kaczmarek, Michalczyk and Diduszko 2005). The new species differs specifically from:

1. *Paramacrobiotus alekseevi*, known only from type locality in Thailand^82^, by: the presence of pores inside egg areoles, a higher *pt* value of second macroplacoid (*15.4–19.8* in the new species vs *9.8–14.5* in *Pam. alekseevi*) and a longer microplacoid (3.3–5.1 μm [*pt* = *6.7–9.3*] in the new species vs 1.9–3.0 μm [*pt* = *4.0–6.2*] in *Pam. alekseevi*). *Remarks:* The comparison was made based on the *Pam. alekseevi* original descripition^77^ as well as the amended description by Stec et al.^71^.
2. *Paramacrobiotus filipi*, known only from type locality in Malaysian part of Borneo^57^, by: the absence of dorsal cuticle granulation, a longer second macroplacoid (7.0–11.3 μm [*pt* = *15.4–19.8*] in the new species vs 2.4–6.2 μm [*pt* = *8.0–13.8*] in *Pam. filipi*), a higher *pt* value of macroplacoid row (*60.7–69.8* in the new species vs *44.4–58.6* in *Pam. filipi*), a longer placoid row (34.9–53.6 μm [*pt* = *77.7–87.4*] in the new species vs 17.4–34.5 μm [*pt* = *52.9–73.6*] in *Pam. filipi*) and a larger full egg diameter (104.8–125.3 μm in the new species vs 99.0–104.5 μm in *Pam. filipi*).
3. *Paramacrobiotus garynahi*, known only from type locality in Baikal Region (Russia)^78^, by: medioventral tooth in the third band of teeth in the oral cavity divided, eggs chorion ornamentation of the *richtersi* type, i.e. areoles with pores inside (*areolatus* type with areoles wrinkled inside in *Pam. garynahi*), a higher *pt* value of macroplacoid and placoid rows (*60.7–69.8* and *77.7–87.4*, respectively, in the new species vs *44.4–56.9* and *55.3–70.3*, respectively, in *Pam. garynahi*) and a smaller eggs bare and full diameter (64.3–91.7 μm and 104.8–125.3 μm, respectively, in new thespecies vs 96.0–132.0 μm and 142.0–180.0 μm, respectively, in *Pam. garynahi*).

### Diversity and distribution of *Paramacrobiotus* taxa

Studies on the genus *Paramacrobiotus* become easier due to several revisions and redescriptions of *Paramacrobiotus* species which have been recently published (e.g.^12,14,15^). The barrier for tardigrade diversity studies is currently being broken down especially by an integrative approach implemented into taxonomic and faunistic research. The tight link between genetic data and the exact specimen/species name and its morphology provided by authors of integrative studies is and will be crucial to understand species diversity in the genus *Paramacrobiotus*. Similarly, to Stec et al.^15^, our molecular analyzes showed 9–10 taxa without a certain assignment to any nominal *Paramacrobiotus* species. They may constitute already known species that were described in the past based on morphology only and for which genetic data are lacking or they constitute new for science species avaiting their formal descriptions. Although the results indicate considerable diversity that is still hidden within the genus, it should be also noted that in our study more putative species were delimited by tree-based methods compared with distance-based methods. However, this finding is in line with recent studies on tardigrades, but also studies on other animal groups^85–87^. Based on the research which examined numerous *Paramacrobiotus* populations^14,15^, we can notice that many species in this genus (especially in the *Pam. richtersi* group) are extremely similar to each other often exhibiting a considerable intraspecific variation in egg chorion morphology. This makes many of these taxa as sutiable candidates to be considered as cryptic or pseudocryptic species^14,15^. Therefore, it seems very likely that future taxonomic studies on the genus *Paramacrobiotus* would be able to formally name many newly discovered evolutionary lineages only by rigorous tests of distinct species hypotheses with integrative methods.

Over the years, species of *Paramacrobiotus* have been recorded in various geographic regions. Nominal species of the genus have been found in six continents (Table 4). Additionally, there are many unconfirmed taxa from *Pam. richtersi* and *Pam. areolatus* group which are known from numerous localities around the world (see e.g.^14,15,26,88–92^). Importantly, verification of these records is now extremely hard and, in many cases, not possible because of the lack of genetic data and original material. The majority of the *Paramacrobiotus* species are known only from their type localities or from very restricted geographic ranges. However, some of them are reported from slightly wider geographical areas, like: *Pam. danielae* from Ecuador and Peru, and *Pam. sklodowskae* from Cyprus and Tunisia. There are also much wider distributed species, like, for example *Pam. centesimus,* known from Brazil and Ecuador, *Pam. gerlachae* from Costa Rica and the Seychelles, *Pam. tonolli* known from Canada and many states in USA or *Pam. vanescens* reported from the Democratic Republic of the Congo, the Republic of Guinea-Bissau, the Republic of Zambia and Tanzania^15,88–91^. However, the most widely distributed species in the genus *Paramacrobiotus*, which should be considered as truly cosmopolitan, is the parthenogenetic *Pam. fairbanksi* reported alredy from Antarctica, Italy, Poland, Spain and USA (Alaska)^28^. Furthermore, parthenogenetic *Pam. gadabouti* sp. nov. described here has been confirmed in our study to be present in Australia, France, Portugal and Tunisia (see Figs. 1 and 2). This, all together with *Pam. fairbanksi*, corroborate that at least some tardigrade species conform to “everything is everywhere” hypothesis. In contrast, other species from the *Pam. richtersi* group which are bisexual, in most cases the range seems to be limited and restricted e.g. *Pam. experimentalis* reported only from Madagascar, *Pam. metropolitanus* from Japan, *Pam. celsus*, *Pam. depressus* and *Pam. spatialis* reported only from Italian locations, but type species of the genus *Pam. richtersi* is reported from Ireland and Finland^14,15,20,56,59^. Importantly when comparing haplotype networks presented for *Paramacrobiotus* taxa in Guidetti et al.^14^ and haplotype network provided in our study (Fig. 2) one may see that the divergence between haplotypes in bisexual species (*Pam. richtersi*, *Pam. celsus*, *Pam. arduus*, *Pam. depressus* and *Pam. spatialis*) seems to be higher than divergence between haplotypes in parthenoegenetic species (*Pam. fairbanksi*, *Pam. gadabouti*). Unfortunately, it is premature to conclude if this result could be considered an actual biological pattern or if it simply reflects biases in the analysed data sets, that might be potentially caused by not very large number of sequences analysed per each studied species/population.

**Table 4:** Type localities of all known *Paramacrobiotus* species.

Geographic region	Total number of species	Type localities and species
Australia and New Zealand	2	a) Australia: *Pam. peteri*^93^; b) New Zealand: *Pam. hapukuensis*^94^
Central America	2	a) Costa Rica: *Pam. Huziori*^80^ and *Pam. Magdalenae*^80^
North America	3	a) USA: *Pam. fairbanksi*^19^, *Pam. halei*^95^ and *Pam. tonollii*^18^
Africa	7	a) Kenya: *Pam. kenianus*^19^; b) Madagascar: *Pam. experimentalis*^20^; c) Republic of Guinea-Bissau: *Pam. priviterae*^96^; d) Seychelles: *Pam. corgatensis*^97^* Pam. danielisae*^98^ and *Pam. gerlachae*^99^; e) Tanzania: *Pam. vanescens*^100^
Asia	7	a) India: *Pam. chieregoi*^101^; b) Japan: *Pam. metropolitanus*^59^; c) Malaysia: *Pam. filipi*^57^; d) New Guinea: *Pam. wauensis*^102^; e) Palau: *Pam. palaui*^19^; f) Sri Lanka: *Pam. savai*^103^; g) Thailand: *Pam. alekseevi*^82^
South America	8	a) Brazil: *Pam. centesimus*^104^; b) Colombia: *Pam. derkai*^77^, *Pam. lachowskae*^58^, and *Pam. sagani*^105^; c) Ecuador: *Pam. danielae*^96^ and *Pam. spinosus*^12^; d) Peru: *Pam. intii*^71^; e) Uruguay: *Pam. rioplatensis*^106^
Europe	15	a) Austria: *Pam. submorulatus*^107^; b) Belarus: *Pam. klymenki*^108^; c) Cyprus: *Pam. sklodowskae*^81^; d) Hungary: *Pam. csotiensis*^107^; e) Greece: *Pam. beotiae*^109^; f) Ireland: *Pam. richtersi*^16^; g) Italy: *Pam. arduus*^14^, *Pam. celsus*^14^, *Pam. depressus*^14^, *Pam. pius*^110^ and *Pam. spatialis*^14^ h) Norway: *Pam. areolatus*^17^ i) Russia: *Pam. garynahi*^77^, *Pam. lorenae*^111^ and *Pam. walteri*^112^

*Paramacrobiotus gadabouti* sp. nov. is the fourth tardigrade species known from more than one zoogeographic realm and the third known from both the Palaearctic and the Australasian realms. The first two being *Echiniscus testudo*^83^ (Doyère 1840) and *Milnesium inceptum*^113^ Morek, Suzuki, Schill, Georgiev, Yankova, Marley and Michalczyk 2019. This is all in line with the recent study on global distribution of the *Minesium* populations which demonstrated that most of the species have limited distribution; however, some others can be considered cosmopolitan^114^. These examples also confirm the hypothesis presented by Guidetti et al.^14^ that parthenogenetic tardigrades should have a wider distribution due to the adventage in inhabiting new places caused by asexual reproduction. On the other hand, it must be noted that most records of these four disuscussed parthenogenetic species (*Ech. testudo*, *Mil. inceptum*, *Pam. fairbanksi*, *Pam. gadabouti* sp. nov.) come from highly populated and often touristic places. Therefore, it is also likely that their wide distribution range was additionally enhanced by human-mediated dispersion^15^ or other vectors such as wind, mammals, birds and animals as evidence has been brought to light regarding the dispersal of tardigrades via these various other vectors^115–117^.

## Supplementary Information

Supplementary Information 1.

Supplementary Information 2.

Supplementary Information 3.

## Data Availability

The datasets generated and/or analysed during the current study are available in the GenBank repository, ACCESSION NUMBER OP394113–OP394114, OP394209–OP394212. The data of all sequences are available for public access.
