# Supplementary material for: Integrative taxonomy reveals new, widely distributed tardigrade species of the genus Paramacrobiotus (Eutardigrada: Macrobiotidae)
Source: Sci Rep. 2023 Feb 7;13:2196. doi: 10.1038/s41598-023-28714-w (PMC9905614; doi:10.1038/s41598-023-28714-w)
Supplement: Supplementary file 1 — Supplementary Information 1. [file 41598_2023_28714_MOESM1_ESM.pdf]

Super Clade II Phylogeny

Mr Bayes

Best partitioning scheme

Scheme Name : start_scheme

Scheme lnL : -23012.6773071

Scheme AIC : 46331.3546143

Number of params : 153

Number of sites : 2950

Number of subsets : 6

Subset	Best Model	# sites	subset id	Partition names
1	GTR+I+G	994	2889c2fb1bf9f0caa22cc409057d23b1	18S
2	GTR+I+G	811	bfd3f838ea65ae9d114a3ad078eff508	28S
3	GTR+G	220	8a568435a1eacd0757843555505a11263	COI_pos2
4	SYM+I+G	219	4678c624c3e9ba568220c1842f1eb93d	COI_pos3
5	GTR+I+G	219	2a109f3301b5e0638b9230c437f23295	COI_pos1
6	K80+I+G	487	6a2d17c31cf1c70a30d027d73033e9b8	ITS2

Maximum Likelihood:

Best partitioning scheme

Scheme Name : start_scheme

Scheme lnL : -23009.5776978

Scheme AIC : 46347.1553955

Number of params : 164

Number of sites : 2950

Number of subsets : 6

Subset	Best Model	# sites	subset id	Partition names
1	GTR+I+G	994	2889c2fb1bf9f0caa22cc409057d23b1	18S
2	GTR+I+G	811	bfd3f838ea65ae9d114a3ad078eff508	28S

3	GTR+I+G	220	8a568435a1eacd075784355505a11263	COI_pos2
4	GTR+I+G	219	4678c624c3e9ba568220c1842f1eb93d	COI_pos3
5	GTR+I+G	219	2a109f3301b5e0638b9230c437f23295	COI_pos1
6	GTR+I+G	487	6a2d17c31cf1c70a30d027d73033e9b8	ITS2

Scheme Description in PartitionFinder format

Scheme_start_scheme = (18S) (28S) (COI_pos2) (COI_pos3) (COI_pos1) (ITS2);

COI Paramacrobiotus Phylogeny

Maximum Likelihood:

Best partitioning scheme

Scheme Name : start_scheme

Scheme lnL : -7977.70068359

Scheme AICc : 16502.7892982

Number of params : 193

Number of sites : 658

Number of subsets : 3

Subset	Best Model	# sites	subset id	Partition names
1	GTR+I+G	220	787c222c3fd320008b879c06120a62c3	Gene1_pos1
2	GTR+I+G	219	fa4964d2cabb811ad402226154a49982	Gene1_pos2
3	GTR+I+G	219	221348b558ce91aa7bdc154b4945821e	Gene1_pos3

Scheme Description in PartitionFinder format

Scheme_start_scheme = (Gene1_pos1) (Gene1_pos2) (Gene1_pos3);

Saturation test with DAMBE

ITS-2 alignment

Test of substitution saturation (Xia et al. 2003; Xia and Lemey 2009)

Analysis performed on all sites.

Testing whether the observed Iss is significantly lower than Iss.c.
IssSym is Iss.c assuming a symmetrical topology.
IssAsym is Iss.c assuming an asymmetrical topology.

NumOTU	Iss	Iss.cSym	T	DF	P	Iss.cAsym	T	DF	P
4	0,626	0,795	5,150	486	0,0000	0,760	4,106	486	0,0000
8	0,698	0,750	1,178	486	0,2396	0,639	1,325	486	0,1857
16	0,782	0,718	1,121	486	0,2629	0,509	4,801	486	0,0000
32	0,870	0,701	2,437	486	0,0152	0,376	7,121	486	0,0000

Note: two-tailed t-tests are used.

Interpretation of results:

	Significant Difference: Yes	Significant Difference: No
Iss < Iss.c	Little saturation	Substantial saturation
Iss > Iss.c	Useless sequences	Very poor for phylogenetics

COI alignment

Test of substitution saturation (Xia et al. 2003; Xia and Lemey 2009)

Analysis performed on all sites.

Testing whether the observed Iss is significantly lower than Iss.c.
IssSym is Iss.c assuming a symmetrical topology.
IssAsym is Iss.c assuming an asymmetrical topology.

NumOTU	Iss	Iss.cSym	T	DF	P	Iss.cAsym	T	DF	P
4	0,370	0,805	18,372	657	0,0000	0,774	17,058	657	0,0000
8	0,385	0,766	13,335	657	0,0000	0,656	9,496	657	0,0000
16	0,407	0,744	10,319	657	0,0000	0,535	3,896	657	0,0001
32	0,428	0,718	7,937	657	0,0000	0,392	0,987	657	0,3239

Note: two-tailed t-tests are used.

Interpretation of results:

	Significant Difference: Yes	Significant Difference: No
Iss < Iss.c	Little saturation	Substantial saturation
Iss > Iss.c	Useless sequences	Very poor for phylogenetics
